# Injectable In-Situ Forming Depot Based on PLGA and PLGA-PEG-PLGA for Sustained-Release of Risperidone: In Vitro Evaluation and Pharmacokinetics in Rabbits

**DOI:** 10.3390/pharmaceutics15041229

**Published:** 2023-04-13

**Authors:** Seyedeh Nesa Rezaeian Shiadeh, Farzin Hadizadeh, Elham Khodaverdi, Mahmoud Gorji Valokola, Saleh Rakhshani, Hossein Kamali, Ali Nokhodchi

**Affiliations:** 1 Department of Pharmaceutics, School of Pharmacy, Mashhad University of Medical Sciences, Mashhad 9177948974, Iran; 2 Biotechnology Research Center, Pharmaceutical Technology Institute, Mashhad University of Medical Sciences, Mashhad 9177948974, Iran; 3 Targeted Drug Delivery Research Center, Pharmaceutical Technology Institute, Mashhad University of Medical Sciences, Mashhad 9177948974, Iran; 4 Department of Pharmacodynamics and Toxicology, School of Pharmacy, Mashhad University of Medical Sciences, Mashhad 9177948974, Iran; 5 Lupin Pharmaceutical Research Center, 4006 NW 124th Ave., Coral Springs, Florida, FL 33065, USA; 6 Pharmaceutics Research Laboratory, School of Life Sciences, University of Sussex, Brighton BN1 9QJ, UK

**Keywords:** risperidone, PLGA, PLGA-PEG-PLGA, Risperdal CONSTA^®^, ISFI, ISFG

## Abstract

In the current research, novel drug delivery systems based on in situ forming gel (ISFG) (PLGA-PEG-PLGA) and in situ forming implant (ISFI) (PLGA) were developed for one-month risperidone delivery. In vitro release evaluation, pharmacokinetics, and histopathology studies of ISFI, ISFG, and Risperdal CONSTA^®^ were compared in rabbits. Formulation containing 50% (*w*/*w* %) of PLGA-PEG-PLGA triblock revealed sustained release for about one month. Scanning electron microscopy (SEM) showed a porous structure for ISFI, while a structure with fewer pores was observed in the triblock. Cell viability in ISFG formulation in the first days was more than ISFI due to the gradual release of NMP to the release medium. Pharmacokinetic data displayed that optimal PLGA-PEG-PLGA creates a consistent serum level in vitro and in vivo through 30 days, and histopathology results revealed nearly slight to moderate pathological signs in the rabbit’s organs. The shelf life of the accelerated stability test didn’t affect the results of the release rate test and demonstrated stability in 24 months. This research confirms the better potential of the ISFG system compared with ISFI and Risperdal CONSTA^®^, which would increase patients’ compliance and avoid problems of further oral therapy.

## 1. Introduction

Risperidone is an antipsychotic drug that is a derivative of benzisoxazole, and currently, it is the most frequently prescribed antipsychotic drug in the United States. It can cause changes in the neurotransmitters of the brain. It has also been confirmed effective in treating autism [1]. An injectable sustain-release formulation of risperidone is an alternative for oral drug delivery, which can provide high patient compliance for patients suffering from psychotic diseases.

Risperidone loaded in non-biodegradable polyurethane-PLGA thermoplastic implants was designed by Endo & Braeburn Pharmaceuticals for the maintenance six-month treatment of schizophrenia. The efficacy and safety of these formulations are being investigated in Phase III of the clinical trial [2]. The application of implantable matrix is restricted owing to side effects in the injection site, such as inflammation and fibrosis as a result of foreign-body reaction [3,4]. Risperdal CONSTA^®^ is a well-known commercial form consisting of 12.5, 25, 37.5, and 50 mg of risperidone combined within microspheres. This formulation is provided as a dry powder along with an aqueous diluter, and an injectable suspension is prepared just before use through intramuscular (IM) injection. Risperdal CONSTA^®^ showed a three-week delay in drug release and needed an injection every two weeks for the first injection. Furthermore, in the in-situ forming implant (ISFI) of risperidone (Perseris^®^), 90 and 120 mg of risperidone is incorporated into the PLGA poly D,L (lactide-co-glycolide); 80:20 molar ratio of lactide to glycolide polymer and N-Methyl-2 pyrrolidone (NMP). As soon as Perseris^®^ is injected subcutaneously (SC), the solvent (NMP) enters the aqueous phase and is replaced by water to form an implant (solvent-induced phase inversion) [5]. Perseris^®^ and Eligard^®^ injections are prepared with two coupled syringes. The initial burst release is observed in this vehicle due to the rapid withdrawal of the solvent; this problem leads to an allergic reaction at the injection site and side effects of the drug. To reduce the initial burst release, it may be reasonable to prepare formulations that interact more with solvent and polymer and leave the vehicle later [6,7,8].

Lately, to overcome the initial burst release, lipid liquid crystals based amphiphilic materials, such as sorbitan monooleate (SMO) [9], glycerol monooleate (GMO) [10], glycerol dioleate (GDO) [11] and glycerol trioleate (GTO) [12] were used as a potential bioactive carrier for depot delivery of several active pharmaceutical ingredients (API). Various studies assessed model hydrophilic and hydrophobic drugs, such as risperidone [11], clonazepam [13], glucose [14] and paclitaxel [15], to be delivered via this depot approach. Liquid crystal systems are an intermediate state of conventional solids and liquids. They are critical to the entity of life as many essential parts of living organisms; likewise, biochemical fluids and cell walls are liquid crystalline in nature [16].

This study aims to improve the formulation of risperidone in the form of in-situ forming gels (ISFG) based on PLGA-PEG-PLGA triblock instead of ISFI based on PLGA, which can release the drug for a month or longer and mainly decrease the initial burst release. It seemed that the hydrogen bonding among the solvent (NMP) and the PEG sections could prevent the quick release of NMP to the surrounding media. In addition, PLGA-PEG-PLGA triblock is a thermosensitive polymer, and it can be influential in matrix formation. Lower initial burst release resulted from a reduction in the NMP outflow rate. The PLGA-PEG-PLGA triblock is liquid at 25 °C, but it turns into a highly viscous fluid as soon as it is injected into the body (37 °C). The gel forms at the injection site by the thermosensitive properties of the triblock and the phase inversion. The drug-triblock solutions are applied directly to the injection site and turned into a gel without surgical procedures [17]. Based on the above explanation, an attempt was made to prepare a sustain-release ISFG and ISFI formulation containing risperidone. The in vitro and in vivo performances of the formulation were compared with the available commercial formulation, Risperdal CONSTA^®^.

## 2. Materials and Methods

### 2.1. Materials

Risperidone was provided by Poursina Company Pharmaceutical Co., Ltd. (Tehran, Iran). Risperdal CONSTA^®^ was purchased from Janssen Pharmaceutical Co. (Raritan, NJ, USA). Poly (lactide-co-glycolide) (PLGA RG 504H, acid terminated, lactide:glycolide 50:50 molar ratio, Mw 38000-54000 Da), poly (lactide-co-glycolide)-block-poly (ethylene glycol)-block-poly (lactide-co-glycolide) (PLGA-PEG-PLGA, lactide:glycolide 50:50 molar ratio, average Mn (1000-1000-1000)) were purchased from Sigma Aldrich (St Louis, MI, USA). NMP was purchased from Merck (Darmstadt, Germany). The mouse fibroblast cell line (L-929) was purchased from Pasteur Institute (Tehran, Iran). Cell culture media (RPMI1640), fetal bovine sera (FBS), Trypsin, and Penicillin-streptomycin are purchased from Gibco (Dreieich, Germany). All other chemicals and reagents purchased from Sigma-Aldrich or Merck were analytical grade.

### 2.2. Preparation of ISFI and ISFG Formulations

PLGA-PEG-PLGA (ISFG 30, 40, and 50 *w*/*w* %) and PLGA (ISFI, 33 *w*/*w* %) were dissolved in NMP (a solvent) containing 50 mg ethyl heptanoate (this solvent can reduce the release of NMP, hence reduces the initial burst release). The solution was then placed in a bath sonicator (25 °C) for 2 h. The formulations were sterilized by autoclave at 121 °C, 3 bar pressure for 20 min. At last, 50 mg of risperidone was added to the sterilized polymeric formulations and sonicated for 1 h to achieve homogenous formulations [18]. The total volume of risperidone powder is always loaded in the vehicle because the mechanism of drug loading is based on the trapping of risperidone into the formulation [19].

### 2.3. Physicochemical Properties of ISFG and ISFI

#### 2.3.1. Syringeability and Rheology

To evaluate the syringeability of the formulations, each formulation was passed through a 20-gauge needle syringe at room temperature at a constant shear rate of 75 S^−1^. In addition, the rheology of ISFG and ISFI was evaluated by cup and cone rheometer (Brookfield, Germany) [20].

#### 2.3.2. Sol-Gel Transition Temperature of ISFG

To specify the phase transition temperature of the copolymer, different concentrations of PLGA-PEG-PLGA (10–50% *w*/*w*) were prepared in phosphate buffer saline (PBS, pH 7.4), and the temperature of the copolymer solution was increased by 0.5 °C per min from 0 to 60 °C under constant stirring (200 rpm). Gelation temperature was noted once the magnetic bar stopped rotating [21].

#### 2.3.3. Scanning Electron Microscopy (SEM)

The prepared formulations, according to Section 2.2, were injected into PBS for three days, and then the samples were freeze-dried for 36 h. The obtained samples were cut, covered with gold (30 mA for 5 min) and placed on the holder [22] to analyze the implant or gel surface and cross-section morphology characteristics by SEM (LEO1450 VP, Zeiss Company, Oberkochen, Germany).

### 2.4. In Vitro Release Assessment

To determine the release profile of ISFI and ISFG, 100 mg of optimized formulations containing 5 mg risperidone were injected into 75 mL PBS as a dissolution medium at 37 ± 0.5 °C via a 20-gauge needle. As the solubility of risperidone is 0.18 mg/mL in PBS, so 75 mL of the aqueous release medium should be able to dissolve the entire drug content (5 mg) if it is released (sink condition) during the test [23]. Immediately after injection into the release medium, the formulation turned into a gel or an implant. The vials containing the release medium were maintained in a reciprocal shaking bath through the test (37 ± 0.5 °C, 100 rpm). At specified time intervals (2, 4, 6, 8, 10, 12, 18, 20, 22, and 24 h; 2, 3, 4, 5, 7, 10, 14, 18, 21, 28, 32 and 35 days), 2 mL of release medium was pulled out and replaced by pure PBS to maintain sink condition.

The withdrawn samples were analyzed by high-performance liquid chromatography (HPLC) (Shimadzu, Japan) to evaluate the risperidone concentration via a calibration curve (3.43–100 ng/ml). LC-20AD pump linked to a diode array detector at 220 nm with a C18 column obtained from SepaChrom, Adamas^®^ (Roma, Italy) (25 cm × 4.6 mm, pore size 100 Å) used in this study. The eluent combined from a blend of 30 (*v*/*v* %) acetonitrile (MeCN), 70 (*v*/*v* %) 0.05 M dipotassium hydrogen orthophosphate (K_2_HPO_4_) (including triethylamine 0.3% *v*/*v*) adjusted to pH 3.7 with orthophosphoric acid (H_3_PO_4_) with a flow rate 0.6 mL/min [24]. The limit of detection (LOD) and limit of quantification (LOQ) were determined based on the signal-to-noise ratio as LOD (3:1) and LOQ (10:1).

Simulation of drug release profiles obtained from ISFG, ISFI, and Risperdal CONSTA^®^ was evaluated by Zero-order, Higuchi, Quadratic, Hixon-Crowell, Korsmeyer-Peppas, and Weibull models using DDsolver software [25,26].

### 2.5. In Vitro Degradation Assessment

To investigate the direct in vitro degradation of PLGA and PLGA-PEG-PLGA without risperidone in distilled water, a weight loss test was carried out at time intervals of 1, 2, 3, 4, 5, 7, 10, 14, 18, 21, 25, 28, 31 and 35 days at human body temperature and 120 rpm. At the predetermined times mentioned above, water was eliminated, and the samples were freeze-dried for 48 h to remove the residual water [27]. The weight loss percentage was calculated according to Equation (1).

(1) $$\mathrm{Degradation}\;\%=\frac{W_{i}-W_{t}}{W_{i}}\;\times \;100$$

W_i_: the initial weight; W_t_: the final weight.

### 2.6. In-Vitro Solvent Exchange Assessment

In vitro NMP release was evaluated via quantifying the total of NMP that was released from the formulations into the release media by HPLC at λ 220 nm, C18 column (Brisa LC2, 4.6 × 250 mm, 5 μm), injection volume 20 μL, and flow rate 0.6 mL/min at room temperature. An isocratic mixture of trifluoroacetic acid (0.1% *v*/*v*) (68% *v*/*v*) and acetonitrile (32% *v*/*v*) was used as eluent with a flow rate of 0.5 mL/min as mobile phase [28,29].

### 2.7. In Vitro Cytotoxicity Assessment

The colorimetric MTT assay was run for cytotoxicity investigation. Mouse L-929 fibroblast cell line was cultured in RPMI 1640 medium supplied by 10% (*v*/*v*) FBS, 100 IU/mL of penicillin, and 100 mg/mL of streptomycin at 5% CO_2_, 37 °C, and 95% humidity for one day [30]. L-929 was cultured at a 96-well plate (10^4^ cells/well) and treated for 24 h with samples taken at release medium of ISFI, ISFI-risperidone, ISFG, ISFG-risperidone, control, and Risperdal CONSTA^®^ at 0.5, 1, 3, 7, 14, 21, 28, 35, days, and diluted by cell culture medium (50%). In the next step, the upper medium was removed, and MTT solution (5 mg/mL) was added and incubated for 4 h. Then, 150 μL dimethyl sulfoxide (DMSO) was replaced to dissolve violet crystals. At last, to calculate the cell viability percentage, the absorbance of the final taken solution was measured by a microplate reader (Epoch Microplate Spectrophotometer, Biotek, USA) at 570 nm (sample) and 630 nm as a reference wavelength, and it was measured using control group as 100% viability percentage [31].

### 2.8. In Vivo Assessment

#### 2.8.1. Pharmacokinetic Evaluation

Healthy New Zealand male rabbits (2 ± 0.1 kg) were purchased from Pasteur Institute (Tehran, Iran) and kept under standard housing conditions (25 °C and 55% air humidity). The whole stages of the in vivo test were performed based on the rules of the ethics committee of Mashhad University of Medical Sciences (IR.MUMS.PHARMACY.REC.1396.212).

Rabbits were divided into seven groups (each group contained three rabbits): untreated control group (group I), ISFI (SC) (group II), ISFG (SC) (group III), risperidone solution in NMP (SC) (group IV), ISFI with risperidone (SC) (group V), ISFG with risperidone (SC) (group VI) and Risperdal CONSTA^®^ (IM) (group VII). In this experiment, only groups IV-VII received 25 mg of risperidone. The groups which received drug-free formulations were used to compare pathology.

To analyze risperidone in the rabbit serum for pharmacokinetic assessment, 1.0 mL of blood was collected at specific times (2, 4. 8, 12, 18, 24, 28, 32 and 36 h and 2, 3, 4, 7, 10, 14, 17, 21, 24, 28, 30, 32, and 35 days) from rabbit’s ear. The serum was divided via centrifuge and kept frozen (−80 °C) for analysis day by HPLC.

To obtain the calibration graph, ten solutions with various concentrations (3.43–120 ng/mL) of risperidone in methanol were injected into HPLC, and the area under each peak was estimated. To extract risperidone from the serum, 500 µL of serum was added to 1000 μL of diethyl ether, then sonicated for 30 min at human body temperature and centrifuged (10,000 rpm) for 15 min. The upper organic phase was separated and evaporated by passing through a gentle stream of nitrogen [32]. The residues were combined with methanol (1.0 mL) and centrifuged (10,000 rpm for 20 min) to analyze risperidone by HPLC, as mentioned in Section 2.4. Finally, PK solver software [33] by non-compartment analysis method was used to calculate the area under the serum risperidone concentration (AUC_0-t_), half-life (t_1/2_), maximum serum risperidone concentration (C_max_), the time required to reach maximum serum concentration (T_max_) and mean residence time (MRT).

#### 2.8.2. In Vivo Degradation Assessment

To investigate the size of the gel or implant by ISFG and ISFI in subcutaneous tissue, the in vivo degradation test was performed via injection of 1.0 mL of the formulation containing risperidone by passing through a 20 G syringe into the backside of the rabbit’s neck. The rabbits were sacrificed 1, 2, 3, 4, and 5 weeks post-injection, and the injection areas, including the surrounding soft tissues, were dissected to evaluate the outward and size of the gel or implant [34].

#### 2.8.3. Histopathology

For the histopathological assessment, rabbits were divested of food for 12 h and were sacrificed by carbon dioxide suffocation. The skin tissues of rabbits were shaved after the end of the pharmacokinetics study, and the injection sites were detached. Furthermore, the heart, liver, brain, and kidneys were removed. The excised tissues were washed with normal saline, fixed in a 10% neutral buffered formalin solution (Accustain^®^), dried, and enclosed in paraffin. Paraffinized tissue fragments were stained by hematoxylin-eosin (H&E), and lastly, light microscopy (Olympus) was used for any signs of pathological examinations [35].

### 2.9. Stability Test

Optimized PLGA-PEG-PLGA and risperidone powder were sterilized and kept in the pre-filled syringe to be tested in accelerated mode. For the stability test, the authors followed the times set by the ICH (The International Council for Harmonization) guideline (ICH Q1C and Q1F) [36] for new drug substances and products to countries of climatic zones III and IV. Performance, safety, and efficacy are evaluated throughout 0, 3 and 6 months under accelerated storage conditions (temperature: 40 °C ± 2 °C; relative humidity (RH): 75% ± 5%).

### 2.10. Statistical Analysis

In the current study, the data were reported as mean ± standard deviation (SD). One-way analysis of variance (ANOVA) was carried out to show the significance of the data using linear regression, where a *p*-value < 0.05 was considered a significant level (GraphPad Prism version 6 software) [37]. For stability data analysis, two-way ANOVA was used, followed by Tukey’s multiple comparisons tests.

## 3. Results and Discussion

### 3.1. Syringability and Rheology

The syringeability results showed that all vehicles could be easily injected via a 20-gauge syringe at ambient temperature (25 °C). Additionally, the final formulations revealed Newtonian fluid; the viscosity of both vehicles was almost constant with increasing shear stress during the test. The viscosities of ISFG and ISFI formulations were 0.15 and 0.45 Pascal-second (Pa·s), respectively. First, there is a sudden increase in the viscosity graph (Supplementary Figure S1), which indicates the time to reach the desired viscosity.

### 3.2. Sol-Gel Transition Temperature of ISFG

**Figure 1:**
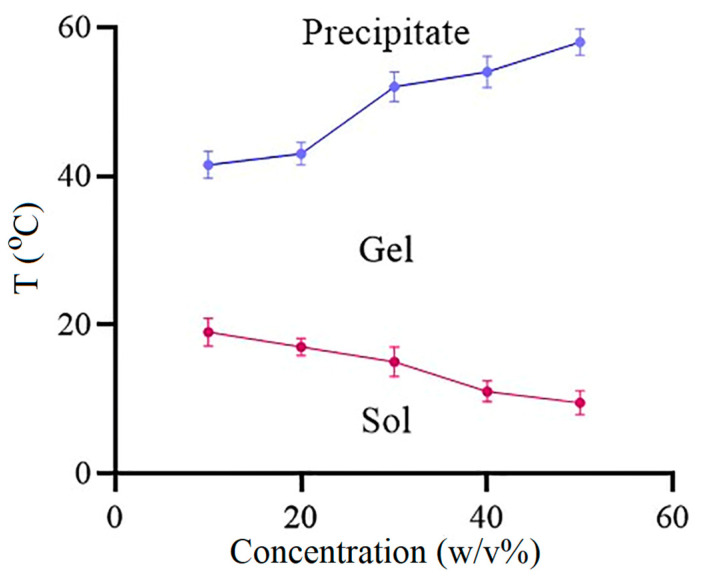
Sol to gel (red) and gel to precipitate (blue) phase diagram of PLGA-PEG-PLGA in water.

The results of the sol-gel test revealed that an elevation in the percentage of copolymer (10–50 *w/v* %) led to an elevation in the precipitation temperature. In addition, it could cause a decrease in sol-gel transition temperature and micellar accumulation acceleration while the percentage of copolymers increases. This is due to the increase in the number of hydrogen bonds followed by an increase in the amount of polymer, which could lead to an elevation of micellar accumulation. The lower sol-to-gel transition temperature leads to faster gel formation and a decrease in initial burst release [38]. In a study carried out by Qiao et al., the gelling temperature decreased with enhancing polymer concentration and significantly intensified by increasing the ratio of lactide to glycolide [39]. The results are presented in Figure 1.

### 3.3. Scanning Electron Microscopy (SEM)

**Figure 2:**
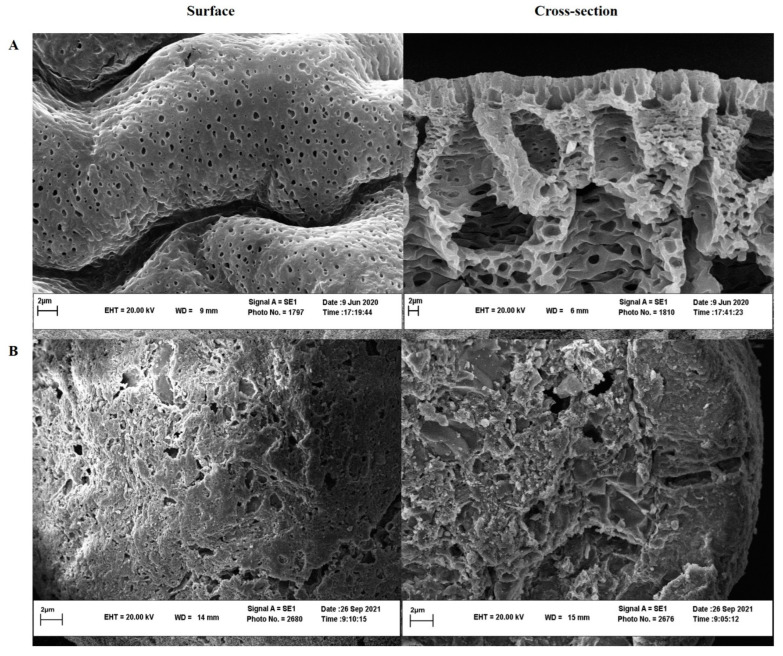
Cross and surface morphologies of ISFI (**A**) and ISFG (**B**) using SEM.

Surface and cross-section SEM images (Figure 2) obtained for ISFI and ISFG showed that ISFI had a porous and spongy texture while ISFG had a less porous structure due to a reduction in solvent outflow at the time of gel formation. It seemed that the hydrogen bonding between the NMP molecules and PEG prevented the rapid diffusion of NMP into the release medium and subsequently left less porosity in the gel. This feature decreases the drug release from the ISFG matrix and subsequently slows initial drug release [40]. In similar studies, the effect of increasing the PLGA polymer concentration on porosity was evaluated [28]. The results showed that the porosity decreased with increasing the polymer concentration in the structure of microparticles. This process may be due to the increase in polymer-polymer interaction concentration and the decrease in the gas penetration from the polymer into the surroundings [41,42]. In addition, in another study performed on PLGA, porosity was examined by adding 4-arm-PEG, and the results indicated that porosity decreased and a more crosslinking degree was obtained [43].

### 3.4. In Vitro Release and Degradation Assessment

**Figure 3:**
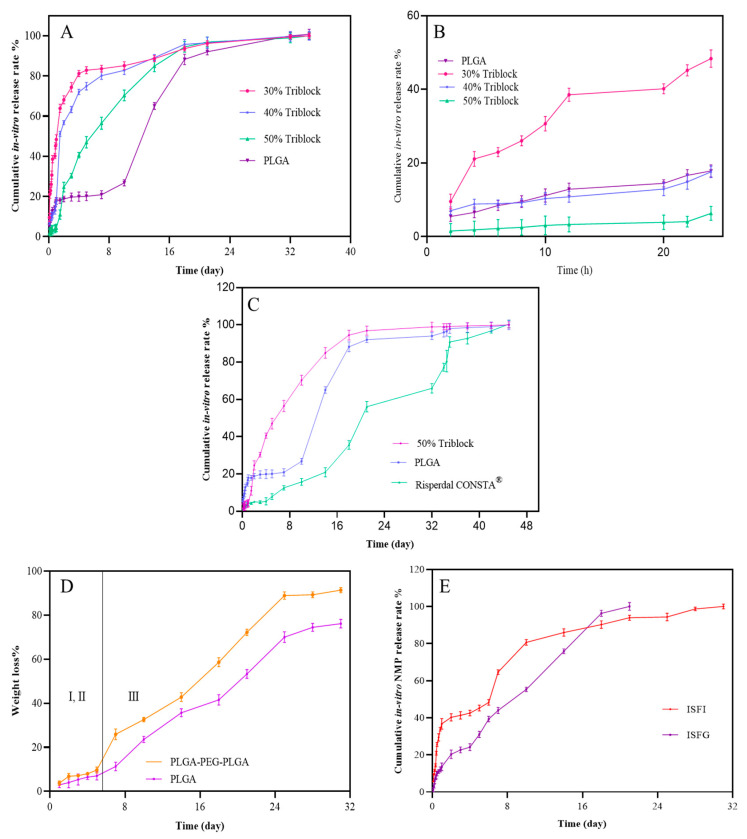
Cumulative in-vitro release percentage of risperidone from ISFG (30, 40, and 50%) and ISFI formulations (**A**), initial in-vitro release percentage (**B**), cumulative in-vitro release percentage of risperidone from optimal tri-block (50%), PLGA, and Risperdal CONSTA^®^ (**C**). In vitro degradation of ISFI and ISFG (**D**) Comparison of the percentage of the cumulative release of NMP from ISFI and ISFG formulations (**E**).

The cumulative in vitro release of risperidone and NMP from ISFI and ISFG (30, 40, and 50%) is revealed in Figure 3A. The initial burst release of risperidone during 24 h from ISFG 30, 40, and 50% were 48.36 ± 2.36%, 17.59 ± 1.55% and 6.33 ± 1.88%, respectively (Figure 3B). Increasing the percentage of tri-block from 30 to 50% leads to enhancing the crosslinks between the copolymer molecules; this elevates the viscosity of the formulations. Additionally, the initial burst release of risperidone from ISFI (17.85 ± 1.652%) was higher than ISFG (PLGA-PEG-PLGA 50%*w*/*w*). The results indicate that ISFG (50%) has the best control of drug release for one month, and the release profile is almost linear. Since, in SEM images, the porosity of the triblock carrier was lower, the ability to control the initial release can be associated with this factor. Furthermore, the drug release profiles of optimum ISFG (PLGA-PEG-PLGA 50%*w*/*w*) and ISFI were compared with Risperdal CONSTA^®^ (Figure 3C). The initial release of Risperdal CONSTA^®^ (4.08 ± 0.27%) was lower than ISFI and ISFG, but this amount is less than the optimal therapeutic limit according to similar studies reported by the Risperdal CONSTA^®^ manufacturer [44]. In resembling studies, lipid liquid crystal carriers using lipids, such as sorbitan mono-oleate and glycerol di-oleate, were used to deliver risperidone; the results showed more initial burst release and a longer release time [11,45].

Kinetics of drug release from the formulation of ISFI during 2–36 h and 46–826 h, tri-block polymers, and Risperdal CONSTA^®^ was evaluated via Zero-order, Higuchi, Quadratic, Hixon-Crowell, Korsmeyer-Peppas, and Weibull models and the results are shown in Table 1 and Supplementary Figures S2–S5. The determination coefficient (R^2^) of the Weibull model was higher for all formulations. According to the Weibull model, the β parameter is effective in the simulation of drug release. A Weibull model with β lower than 0.75 follows the Fick model; if β is between 0.75 and 1 follows the Fick model and drug transfer model II, while for β numbers higher than 1, combined drug release methods are proposed [46]. β number for PLGA and Risperdal CONSTA^®^ is higher than 1, and for tri-block is between 0.75 and 1; therefore, in PLGA and Risperdal CONSTA^®,^ all drug release methods play a role, and for tri-block, there are two methods of Fick model and drug transfer model II.

Figure 3D shows the degradation process of the ISFI and ISFG systems without risperidone based on weight loss in distilled water. The degradation process consists of three phases. In phases I and II, the polymer systems are formed. The exchange of materials occurs with the surrounding phase, but the polymers’ weight is almost constant during this period; in phase III, the degradation of the polymers begins and increases over time. The drug release profile has similar behavior for ISFI and ISFG formulations containing risperidone. The investigation into the degradation of Risperidal Consta^®^ was not carried out. The drug contained within the PLGA microspheres is mostly released through diffusion in a period where the weight of the microspheres should not vary. During this period, the drug has a low release rate from Risperidal Consta^®^ compared to ISFG and ISFI. It is recommended that patients also take oral risperidone concurrently during this time. Subsequently, the microspheres begin to degrade, leading to drug release through a combination of polymer degradation and diffusion. The degradation process begins gradually and then speeds up over time, and this trend is reflected in the drug release profile.

ISFG and ISFI vehicles were degraded within 31 days and lost about 91% and 76% of their weight, respectively. Due to the presence of PEG in the ISFG system, water penetration is higher, and the rate of degradation increases [47]. Therefore, the ISFG system will release the loaded drug faster (within 21 days). In a study conducted by Qiao et al., the degradation percentage was evaluated with different ratios of triblock. They showed that increasing the proportion of d,l-lactide/glycolide from 6 to 15 caused an increase in the degradation time from 15 to 22 days [39]. In a similar study conducted by Milacic and Schwendeman in 2013, the weight loss percentages of PLGA-PEG-PLGA tri-block, di-block copolymer PLGA/PEG blended with PLGA, and PLGA polymer were evaluated. In this study, due to increasing the removal rate of acidic degradation products by PEG, it was therefore expected that the rate of auto-catalytic and mass loss would slow. However, due to the PEG hydrophilicity nature, the water content in the triblock hydrogel tissue was higher than that of the PLGA polymer, and the degradation process was faster [48].

In vitro PLGA-PEG-PLGA thermo-gel degradation test published in 2020 by Chan et al. revealed that the mass of residual thermo-gel reduced from day 15 at a more rapid rate, and the complete gel degradation reached after 35 days [49].

The cumulative release percentage of the NMP in the first 24 h after injection of ISFI formulation (36.7 ± 2.89%) is much higher than the ISFG formulation (13.54 ± 2.08%), which indicates that the ISFG is better than the ISFI for controlling the initial burst release (Figure 3E). Higher solvent release on the first day also leads to high drug release and swelling at the injection site and systemic toxicity [50]. The hydrogen bond of the terminal hydroxyl group of PEG in triblock and the carbonyl group of the NMP solvent can lead the solvent to leave the formulation later and show better control over the initial and long-term risperidone release.

### 3.5. In Vitro Cytotoxicity Assessment

**Figure 4:**
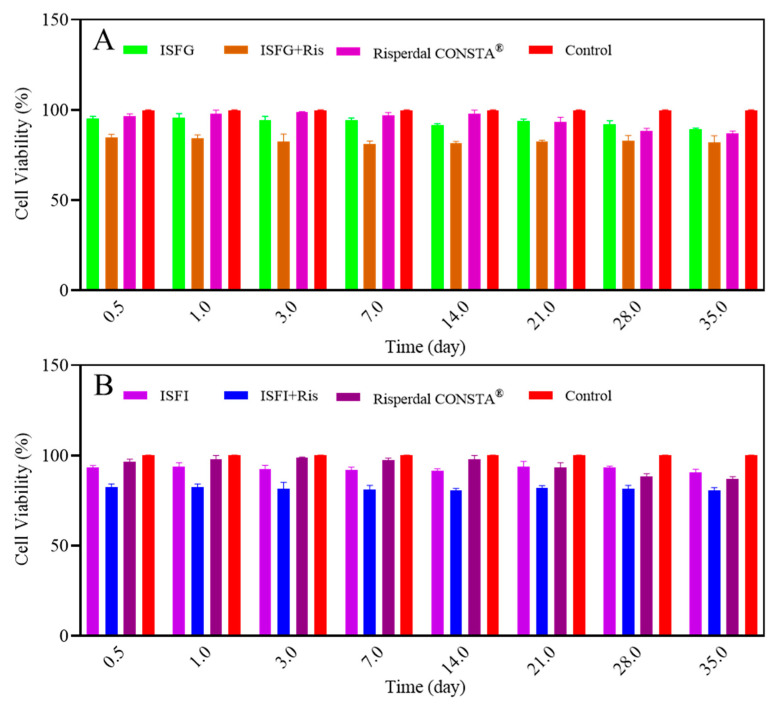
Evaluation of L-929 cell viability in the presence of in vitro release medium of ISFG (**A**) and ISFI (**B**) and Risperdal CONSTA^®^ formulations.

The in vitro toxicity results showed that when ISFG and ISFI without risperidone were employed, the solvent was released on the first day, and the cell viability was slightly lower than in the control group. The same results were obtained for other days between 1 and 35 days (Figure 4). In contrast, in the early days after injection of Risperdal CONSTA^®^, slight cytotoxicity was observed, which may be due to solvent release. Then, after two weeks of injection, minor cytotoxicity was observed in this group due to the release of risperidone. In the groups receiving ISFI and ISFG containing risperidone, an acceptable and uniform level of toxicity (lower than 20% with cell viability) was observed in the early days due to the release of solvents and risperidone while maintaining until the last days (Figure 4).

Cell viability in formulations containing ISFG in the early days was higher than ISFI due to the gradual release of NMP to the release medium. Concentration-toxicity-dependent was observed at all times. At a lower concentration of solvent and polymer, the samples were less toxic (Figure 4). This trend was also observed in the Fischer study, where an increase in the polymer concentration and the duration of contact with L-929 caused a reduction in the percentage of cell viability [51]. In another study carried out by Eroglu et al. (2019), they showed that when different concentrations of ibuprofen were loaded into hydrogel networks and crosslinked PLGA-b-PEG-MA nanoparticles, the cell viability was higher than 80% for different concentrations [52]. Yoshimoto et al., 2018 also showed that when the L-929 cell line was seeded on the PLGA bilayer membrane and evaluated by MTT assay, no change in the number of cells on days 1 and 3 was observed.

### 3.6. In Vivo Assessment

#### 3.6.1. Pharmacokinetic Evaluation

ISFG formulation containing 50% PLGA-PEG-PLGA showed a uniform release for about one month. Therefore, in the current study, triblock 50% and PLGA were compared with Risperdal CONSTA^®^. HPLC chromatogram of risperidone was observed in serum at 3.13 ± 0.12 min, and rabbit blood samples taken at one month at 3.08 ± 0.12 min were shown in Supplementary Figure S6A,B, respectively. A linear calibration curve for concentration vs. absorbance was obtained for risperidone by using HPLC techniques (R^2^ = 0.9983). The LOQ and LOD were 10.29 ± 1.36 and 3.43 ± 0.87 ng/mL for risperidone, respectively.

**Figure 5:**
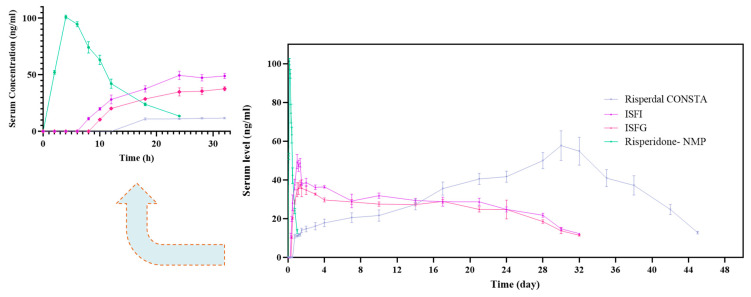
Diagram of blood concentration of risperidone after in-vivo administration of 25 mg risperidone in optimal ISFG, ISFI, risperidone solution in NMP, and Risperdal CONSTA^®^.

Figure 5 demonstrates SC administration of 25 mg of risperidone in NMP, which creates a sudden increase and then, after about 20 h, a sharp decrease in the concentration of risperidone in the rabbits’ blood. In contrast, sustained-release formulations create almost uniform blood levels for approximately one month. Pharmacokinetic data obtained from PK solver software are shown in Table 2. The results of pharmacokinetic data analysis show that the time required to reach the maximum concentration (T_max_) in the ISFG and ISFI groups is 32 ± 6.11 and 24 ± 3.25 h. However, it is 720 ± 6.48 h in the group receiving Risperdal CONSTA^®^, which indicates the capability of ISFI and ISFG to reach a therapeutic concentration in a shorter period. The bug of Risperdal CONSTA^®^ is the delay in drug release of up to about 14 days, so the patient is forced to use oral medication. The maximum concentration of risperidone (C_max_) of ISFG (37.60 ± 1.11 ng/mL) was less than ISFI (49.46 ± 2.82 ng/mL) and Risperdal CONSTA^®^ (57.72 ± 7.62 ng/mL), which indicates the uniform release of the drug from an implant or gel vehicles for one month in comparison to Risperdal CONSTA^®^. The half-life (t_1/2_) in the ISFG and ISFI groups were equal to 169.31 ± 58.08 h and 116.19 ± 48.92 h, respectively, and for the group receiving Risperdal CONSTA^®^, it was 111.42 ±19.81 h, which indicates long-term drug delivery for one month. The area under the curve (AUC) of concentration versus time was 19,246.59 ± 1084.78 ng·h/mL for ISFG and 21,406.59 ± 564.45 ng·h/mL in the case of ISFI, while this value for the commercial formulation was 35,641.73 ± 2840.27 ng·h/mL. Although the mean AUC for ISFG was lower when compared to ISFI, there were no significant differences observed between them. Additionally, mean residence times (MRT), which represent the body’s drug distribution for ISFG, were approximately similar to Risperdal CONSTA^®^.

Local and systemic drug delivery for various drugs has been studied in different parts of the body. In the study of Xie et al., it was observed that Avastin^®^/hydrogel (PLGA-PEG-PLGA) had provided an optimum therapeutic level and stability in the vitreous humor for at least six weeks, while no amount of drug was detected at the injection site with an aqueous solution of Avastin^®^ [53]. In a similar study conducted by Gao et al. in 2011, PLGA-PEG-PLGA tri-block was used for local drug delivery of docetaxel, and the results of intraperitoneal and intravenous (IV) injections showed that IV injection released a high concentration of docetaxel (1978 µg/mL) into the bloodstream in a short time (15 min) and decreased rapidly, but in the intraperitoneal injection of docetaxel loaded in tri-block solution, the amount of drug release (14.03 µg/mL) was much lower (within the therapeutic index) during 25 days of release test [54]. Another study conducted by Cao et al. in 2019 on the effects of sustained-release tri-block copolymer exhibited that the polymer-loaded formulation group produced a consistent level of liposomal doxorubicin during the test days at the tumor site and the final size of the tumors was smaller than other methods of drug delivery [55]. The results of these and similar articles were in line with our study.

#### 3.6.2. In Vivo Degradation Assessment

**Figure 6:**
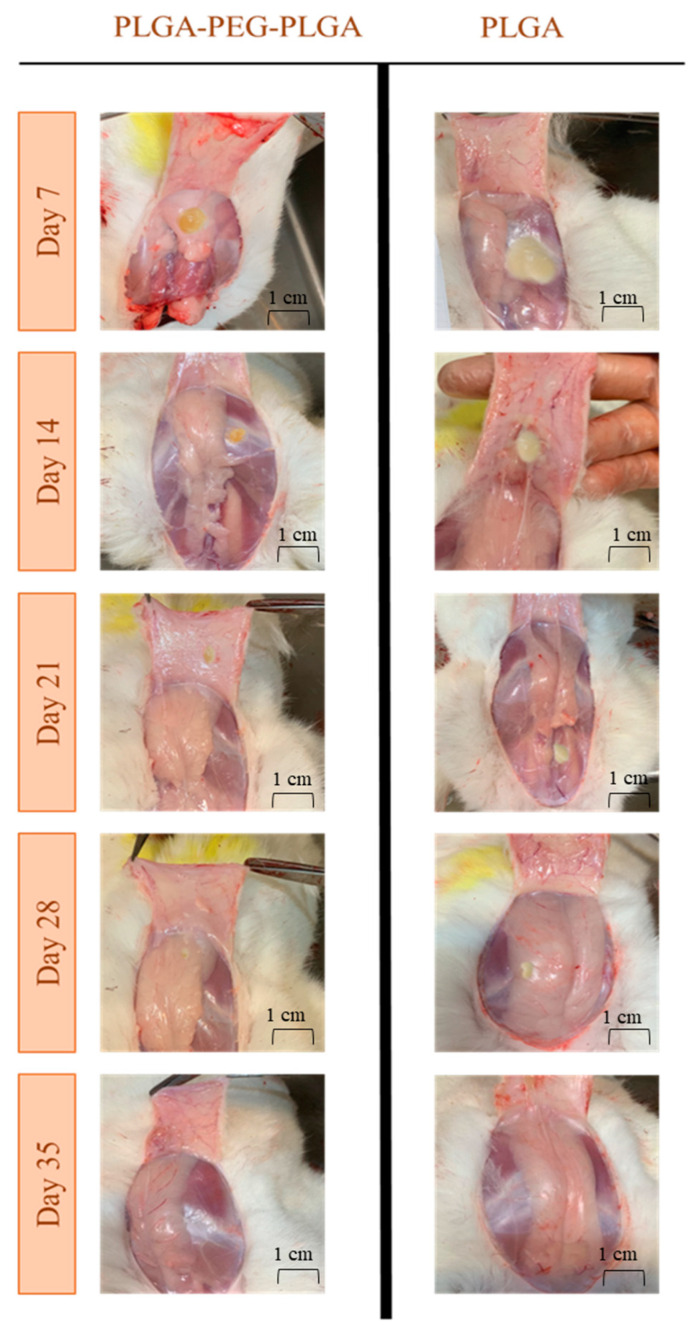
Formation and degradation of ISFG gel and ISFI implant after SC injection into the backside of the rabbit’s neck.

In-situ forming gel and implant formation in the SC region were observed after an autopsy. The ISFG was a light-yellow semitransparent gel-like mesophase, and the ISFI was a light-beige implant (Figure 6). No unusual sign was observed in the SC tissue in all rabbits at 1, 2, 3, 4, and 5 weeks after the injection with the naked eye. Approximately 100% of the ISFG and 85% of the ISFI were degraded entirely within four weeks. These results are consistent with pharmacokinetic data, which indicates that degradation occurs over one month (degradation was traced up to 35 days) and can maintain the blood concentration in the therapeutic index. In a similar study, dexamethasone-loaded PLGA-PEG-PLGA thermo-gel was injected subconjunctival, and the dexamethasone-loaded thermo-gel was twisted to an opaque hard gel. The gel structure remained intact for up to a week and completely vanished after 28 days [49]. In a study conducted by Cao et al. in 2019, the formation of the PLGA-PEG-PLGA-based thermo-gel at the subcutaneous injection site during days 4, 8, and 16 was evaluated. The polymer solution (20 wt%) degraded gradually within 16 days [55].

#### 3.6.3. Histopathology

**Figure 7:**
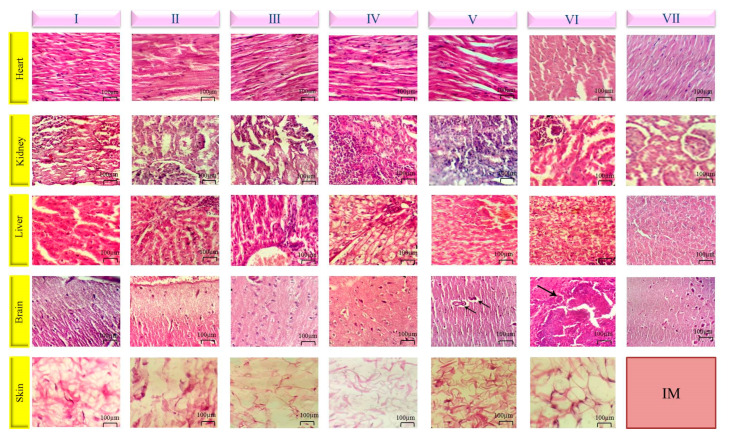
Untreated control group (**I**), ISFI (SC) (**II**), ISFG (SC) (**III**), risperidone solution in NMP (SC) (**IV**), ISFI with risperidone (SC) (**V**), ISFG with risperidone (SC) (**VI**) and Risperdal CONSTA^®^ (IM) (**VII**). The pathological sign is indicated by arrows. H & E staining images × 400.

Figure 7 demonstrates the structure of the histopathological evaluation of heart, kidney, liver, brain, and skin tissues for the seven mentioned groups. No clinical signs were observed in the control and Risperdal CONSTA^®^ groups. Heart tissues in groups treated with risperidone solution in NMP, ISFI-risperidone, ISFG-risperidone, and ISFG were healthy and normal. At the same time, slight inflammatory cell infiltration was assigned in the group treated by ISFI. Cardiotoxicity evaluation in a similar study that used PLGA-PEG-PLGA hydrogel free of the drug showed no cardiac toxicity after the evaluation period [55].

Kidney tissue showed slight necrosis in the groups that received NMP-risperidone and ISFI-risperidone. Additionally, slight to moderate inflammatory cell infiltration was observed in the groups receiving NMP-risperidone, ISFI-risperidone, and ISFG-risperidone. Liver tissue revealed little to mild inflammatory cell infiltration in the portal triads of the groups treated by NMP-risperidone, ISFI, and ISFG. In addition, mild vacuolar degeneration was observed in NMP-risperidone and ISFG groups, and slight necrosis was obtained in ISFG-risperidone and ISFI-risperidone. ISFG-risperidone-treated animals showed slight inflammatory cells in the brain parenchyma, meninges, and necrosis, and also, slight hemorrhage was observed in animals that received ISFI. No sign indicates the pathological problem was detected in the skin of all groups. In this study, slight to moderate toxicities were observed in groups receiving formulations containing risperidone due to the drug or its solvent.

In a relevant article, triblock hydrogel (PLGA-PEG-PLGA) was used for local chemotherapy drug delivery, and at the end of the study, the systemic toxicities were evaluated in the heart, spleen, lung, kidney, and liver tissues; it was also observed that no obvious abnormality was observed in all mentioned organs [56]. In several studies, the effects of PLGA polymer on various organs of laboratory animals were evaluated, and non-toxicity to low toxicity in some organs were the results of these studies. For instance, in the study carried out by Rucker et al., the accumulations of macrophages and polymorphonuclear leukocytes (PMNs) around the PLGA scaffold were observed [57,58].

### 3.7. Stability Test

**Figure 8:**
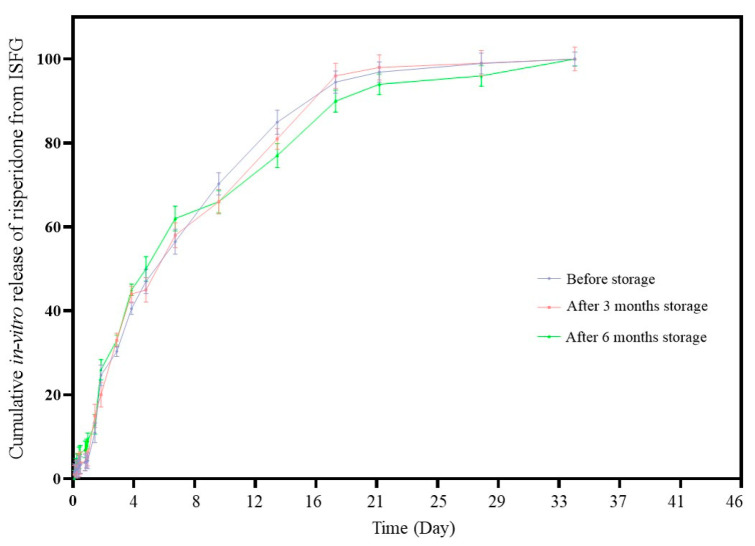
The release profile of ISFG formulation at different times (0, 3rd and 6th months) under accelerated stability test conditions.

The appearance of the formulations showed no change at time points. To evaluate the stability, an in-vitro release test was run after the storage of the formulations for 3 and 6 months and compared with before storage. The results of in vitro release studies of ISFG before and after storage for 3 and 6 months are shown in Figure 8. Stability data were evaluated using Two-way ANOVA statistical analysis by Tukey posttest, and the main effect at the release points in each sample was compared. The difference in the percentage of drug released at each endpoint of the dissolution test was not significant between before and after storage in the stability study (*p*-value = 0.1012). Therefore, it can be concluded that the formulations should be stable for at least 6 months without any changes in the release profiles of the formulation. The difference factor (f1) and similarity factor (f2) showed that all dissolution profiles are similar (f1 value was 7 and 13%, and f2 values were 79% and 71.5% when 3 and 6 months were compared to the release profile before storage, respectively). The similarity factor higher than 50% and the difference factor lower than 15% are an indication of similarity between dissolution profiles. Based on these data, it can be concluded that the accelerated stability conditions can’t affect the rate of drug release in vitro. In this test, mean dissolution times (MDT) were calculated to be 181.5, 183.8, and 194.5 h for 0, 3, and 6 months, respectively. This also indicates that the release profile is stable after storage for 3 and 6 months, as MDT data for different storage times were close to each other.

## 4. Conclusions

The present study demonstrates that the ISFG formulation shows promising potential as a sustained-release system for risperidone. By effectively reducing the initial burst release, the risk of adverse effects associated with high blood concentrations during the first 24 h of treatment can be mitigated. The hydrogen bond between NMP and PEG block in PLGA-PEG-PLGA is suggested to be responsible for the lower initial burst release observed in the ISFG formulation. Moreover, the sustained drug release profile of the ISFG formulation observed in vitro and in vivo for up to one month, within the therapeutic range, highlights its potential as a viable alternative to current treatment options. Importantly, the biocompatibility and biodegradability of the copolymers and NMP solvent used in the formulation offer further advantages over other sustained-release options. Overall, the findings of this study suggest that ISFG formulation could be a valuable addition to the treatment of schizophrenia.

## Figures and Tables

**Table 1:** Regression coefficient (R^2^) of mathematical models.

Model	Equation	Risperdal CONSTA^®^	ISFI 2–36 h	ISFI 46–826 h	ISFG
Zero-order	F = K_o ×_ t	0.9450	0.1675	0.8434	0.5691
Higuchi	F = kH × t^0.5^	0.7554	0.9448	0.8242	0.9184
Quadratic	F = 100 × (k1 × t^2 + k2 × t)	0.9680	0.8841	0.9262	0.9736
Korsmeyer-Peppas	F = kK_p ×_ t^n^	0.961	0.9577	0.8985	0.9184
Hixon-Crowell	F = 100 × [1 − (1 − k_H_C × t)^3^]	0.8783	0.2732	0.9094	0.9911
Weibull	F= 100 × {1 − Exp[-(t-Ti)β/α]}	0.9785	0.9817	0.9613	0.9943

**Table 2:** Pharmacokinetic parameters resulting from the administration of formulations containing 25 mg of risperidone to rabbits (n = 3) using PK solver software.

Groups	AUC_0-t_ (ng h/mL)	T_max_ (h)	C_max_ (ng/mL)	t_1/2_ (h)	MRT (h)
Risperidone solution in NMP (SC) (group IV)	1122.99 ± 46.25	4.00 ± 0.00	101.06 ± 1.69	7.33 ± 0.92	11.95 ± 0.24
ISFI-Risperidone (SC) (group V)	21,406.59 ± 564.45	24.00 ± 3.25	49.46 ± 2.82	116.19 ± 48.9	393.77 ± 130.89
ISFG-Risperidone (SC) (group VI)	19,426.59 ± 1084.78	32.00 ± 6.11	37.60 ± 1.11	169.31 ± 58.08	430.58 ± 58.46
Risperdal CONSTA^®^ (IM) (group VII)	35,641.73 ± 2840.27	720.00 ± 6.48	57.72 ± 7.61	111.42 ± 19.81	635.22 ± 23.37

AUC: Area Under the Curve, T_max_: Time required to reach maximum concentration, C_max_: maximum serum risperidone concentration, t_1/2_: The half-life, MRT: Mean Residence Time.

## Data Availability

The data presented in this study are available on request from the corresponding author.

## Supplementary Materials

The following supporting information can be downloaded at: https://www.mdpi.com/article/10.3390/pharmaceutics15041229/s1. Supplementary Figure S1. Rheology diagram of PLGA-PEG-PLGA (A) and PLGA (B). Supplementary Figure S2. Simulation of drug release profile from the optimal formulation of PLGA 2–36 h. Supplementary Figure S3. Simulation of drug release profile from the optimal formulation of PLGA 46–826 h. Supplementary Figure S4. Simulation of drug release profile from the optimal formulation of tri-block. Supplementary Figure S5. Simulation of drug release profile from the optimal formulation of Risperdal CONSTA^®^. Supplementary Figure S6. HPLC chromatogram of Ris (A) and rabbit blood samples taken at week forth (B).
