# Supplementary material for: Injectable In-Situ Forming Depot Based on PLGA and PLGA-PEG-PLGA for Sustained-Release of Risperidone: In Vitro Evaluation and Pharmacokinetics in Rabbits
Source: Pharmaceutics. 2023 Apr 13;15(4):1229. doi: 10.3390/pharmaceutics15041229 (PMC10143068; doi:10.3390/pharmaceutics15041229)
Supplement: Supplementary file 1 [file pharmaceutics-15-01229-s001.zip › pharmaceutics-2231903-supplementary.pdf]

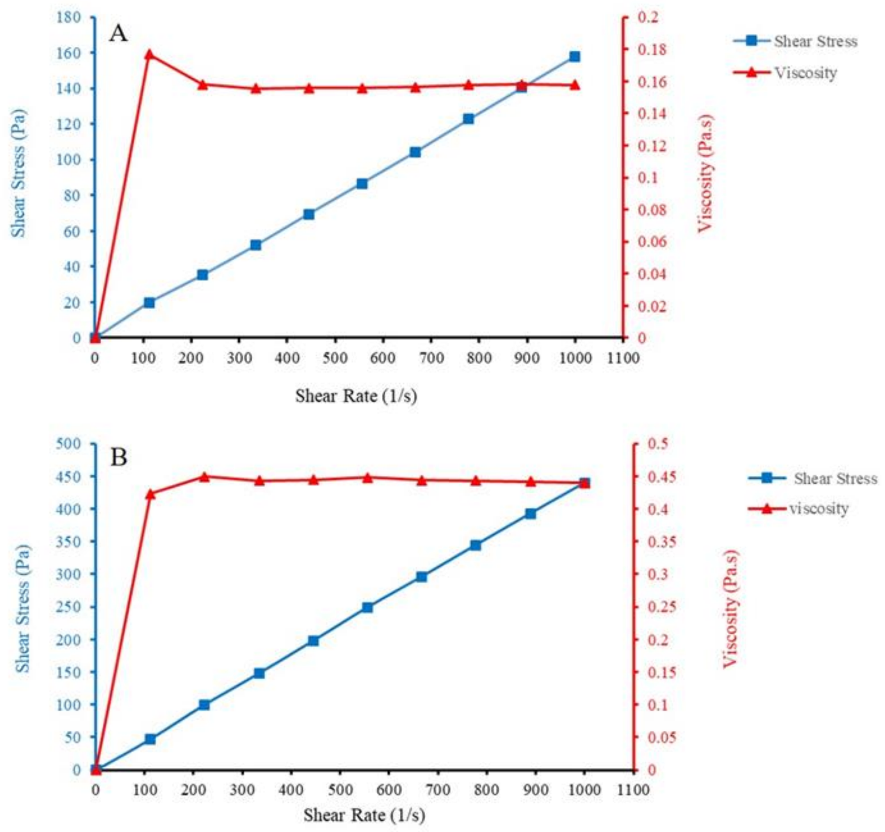

Supplementary Figure S1. Rheology diagram of ISFG (A) and ISFI (B) formulations.

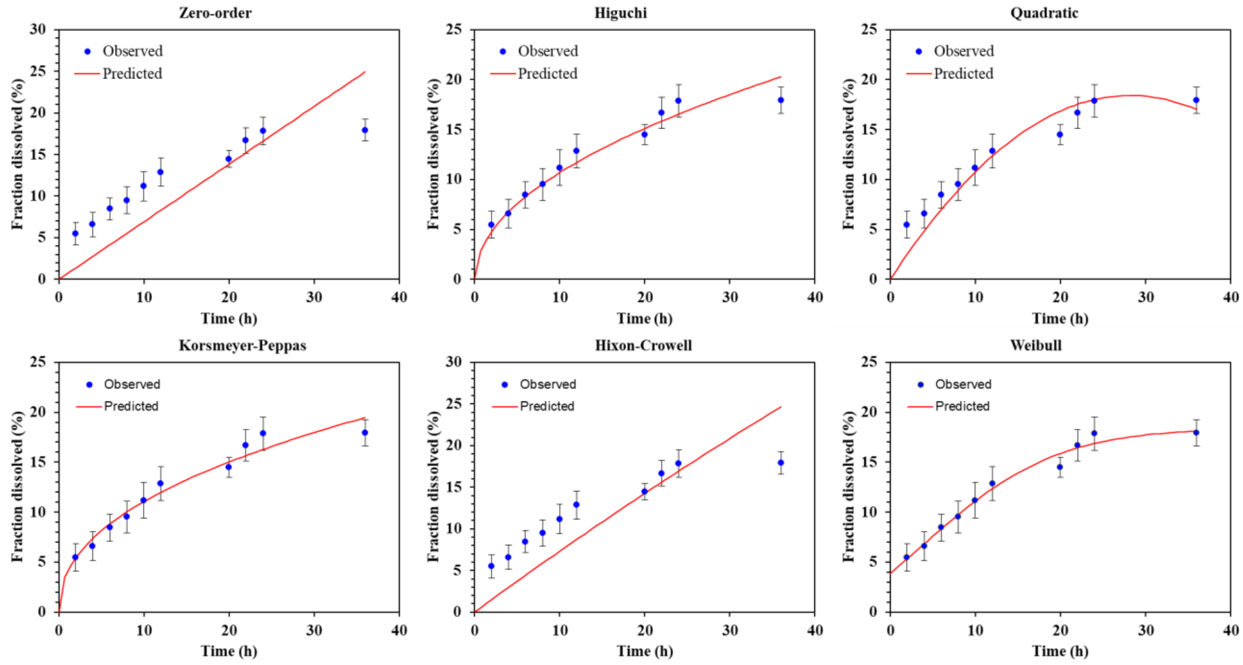

Supplementary Figure S2. Simulation of drug release profile from the optimal formulation of ISFI (2-36 h).

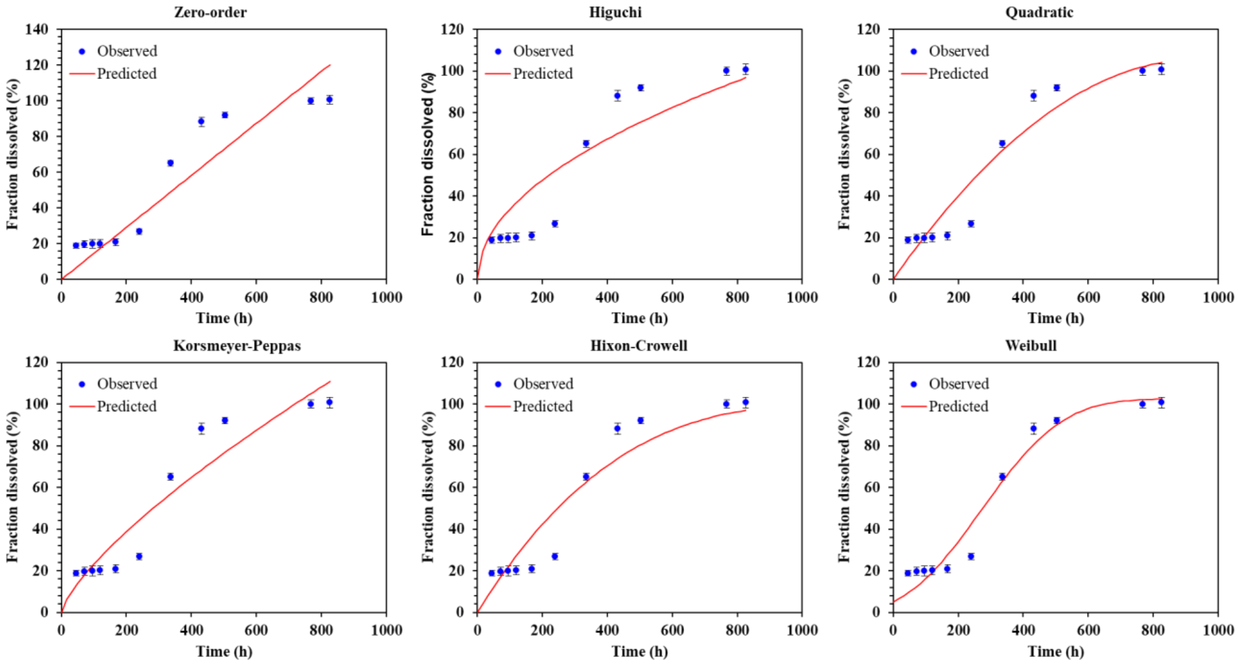

Supplementary Figure S3. Simulation of drug release profile from the optimal formulation of ISFI (46-826 h).

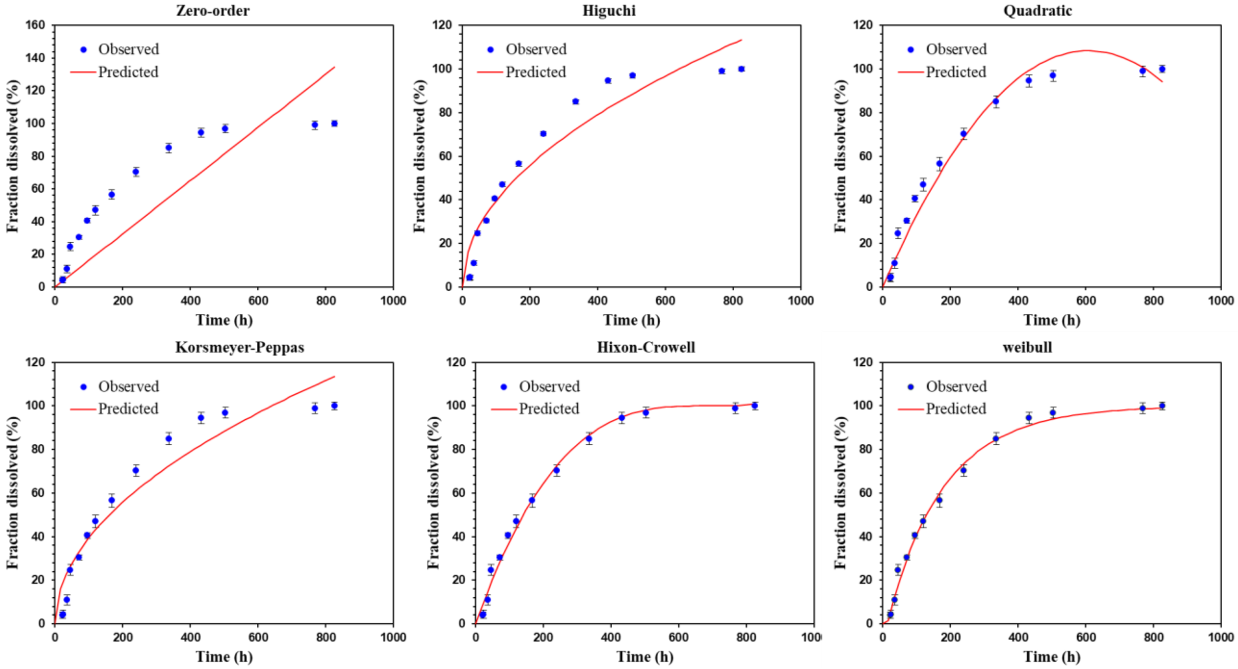

Supplementary Figure S4. Simulation of drug release profile from the optimal formulation of ISFG.

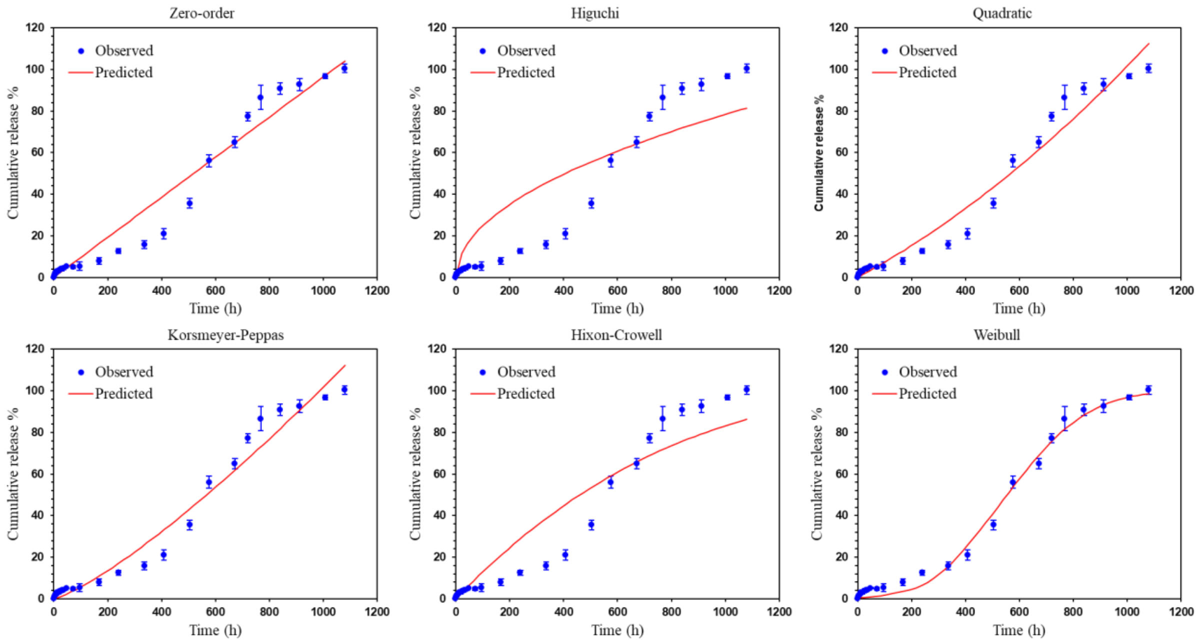

Supplementary Figure S5. Simulation of drug release profile from the Risperdal CONSTA®

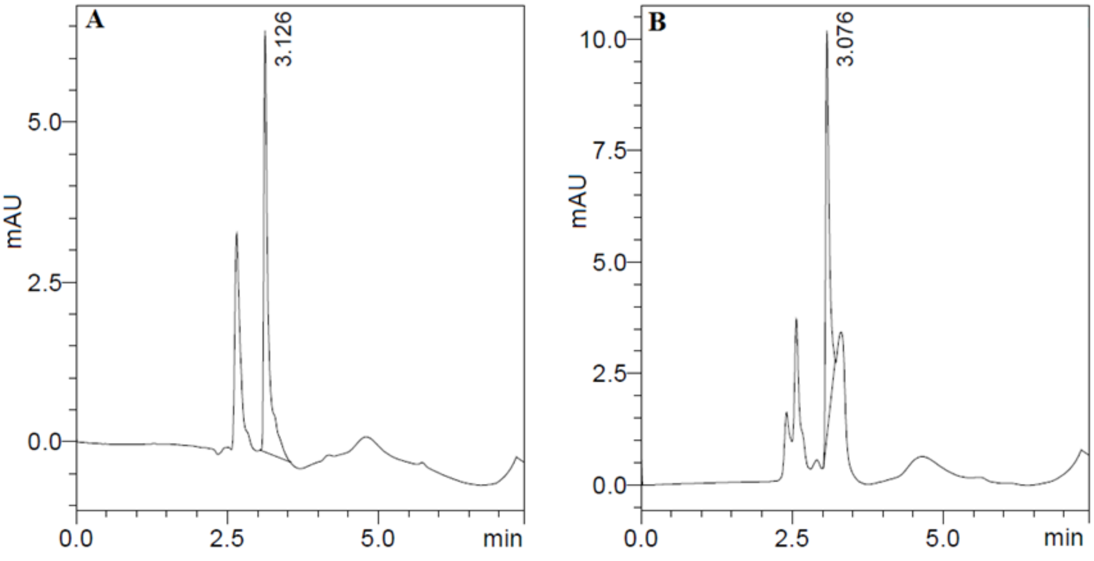

Supplementary Figure S6. HPLC chromatogram of risperidone (A) and rabbit blood samples taken at week forth (B)
